# Pre-pandemic predictors of parental substance use during COVID-19

**DOI:** 10.3389/frcha.2025.1587146

**Published:** 2025-10-07

**Authors:** Christopher J. Greenwood, Primrose Letcher, Delyse M. Hutchinson, Jacqui A. Macdonald, Jasmin Grigg, Joseph M. Boden, John W. Toumbourou, Craig A. Olsson

**Affiliations:** ^1^ SEED Lifespan Strategic Research Centre, Faculty of Health, School of Psychology, Deakin University, Geelong, VIC, Australia; ^2^ Centre for Adolescent Health, Murdoch Children’s Research Institute, Melbourne, VIC, Australia; ^3^ Department of Paediatrics, Royal Children’s Hospital, University of Melbourne, Melbourne, VIC, Australia; ^4^ National Drug and Alcohol Research Centre, Faculty of Medicine, University of New South Wales, Sydney, NSW, Australia; ^5^ Turning Point, Eastern Health, Melbourne, VIC, Australia; ^6^ Monash Addiction Research Centre, Eastern Health Clinical School, Monash University, Melbourne, VIC, Australia; ^7^ Department of Psychological Medicine, University of Otago Christchurch, Christchurch, New Zealand

**Keywords:** families, substance use, COVID-19, machine learning, cohort study

## Abstract

**Aims:**

To examine pre-pandemic predictors of parent substance use during COVID-19 in Australia, where some of the longest periods of public health restrictions in the world occurred.

**Methods:**

We used data from the Australian Temperament Project Generation 3 Study on 560 parents (59% female) who completed COVID-19 specific surveys (2020/2021), including assessment of alcohol, tobacco, and illicit substance use. Comprehensive pre-pandemic assessments were conducted during the postpartum period when offspring turned 1-year of age (2012–2018), including 33 indicators spanning parent and child factors (individual, relational, and contextual).

**Results:**

During the pandemic, 39% of parents reported drinking alcohol 3-to-4 days per week or more, 12% used tobacco, and 6% used illicit substances. In Least Absolute Shrinkage and Selection Operator (LASSO) logistic regression models, a variety of pre-pandemic predictors [k] were identified across alcohol (AUC = 0.72, k = 2; OR = 0.92–2.03), tobacco (AUC = 0.96, k = 10; OR = 0.61–4.21), and illicit substance use (AUC = 0.78, k = 2; OR = 1.44–1.60). The strongest predictors were pre-pandemic use of the same substance (OR = 1.60–4.21). While few other predictors were identified for alcohol and illicit substance use, several family characteristics predicted tobacco use.

**Conclusions:**

Results indicated that parents engaging in alcohol, tobacco and illicit drug use in our cohort reported strong continuity of use from before, to during the COVID-19 pandemic in Australia. This highlights the importance of public health initiatives that provide accessible substance use support and treatment options to parents during periods of public health emergencies. Further, enriched population interventions targeted across socioeconomic and family contexts may be important in identifying risk, particularly for tobacco use.

## 1 Introduction

The COVID-19 pandemic has had a significant impact on population health and wellbeing worldwide. In Australia, strict social distancing and isolation measures were implemented across 2020–2021 to slow the rate of infection (1). While these measures achieved their direct aim of containing virus transmission, they also provoked a cascade of population-wide outcomes including changes to patterns of alcohol, tobacco, and illicit substance use. For example, during the COVID-19 period in Australia, there was an increase in alcohol-induced deaths, coupled with a decrease in drug-induced deaths (2). More broadly, patterns of substance use varied across populations, pointing to considerable heterogeneity in risk for substance use (3, 4) and highlighting the “need to recognise the existence of disadvantage in our response to crises” (5).

One of the hardest hit sectors of society were families with young children, where pandemic-related restrictions radically reduced access to childcare and education services, and introduced new and often complex challenges balancing working from home, with childcare and/or home-schooling demands (6, 7). In this context, potential changes in substance use in the family environment during COVID-19 have received growing attention because of the unique risks that substance use can pose to parents and children (8, 9) including substance-induced intimate partner violence and child maltreatment (10, 11).

Systematic reviews on the COVID-19 context have identified parenting and caregiving responsibilities to be associated with increased substance use, although evidence has primarily focused on alcohol use (3, 4). Further, findings from one Australian sample of parents identified the importance of demographic (e.g., gender, single parent household) and individual (e.g., chronic physical conditions) factors in parental tobacco and alcohol use (12, 13). Nevertheless, further examination of a broad range of factors related to parental substance use during the pandemic, including substances other than alcohol and tobacco, is warranted.

Further to this, to accurately identify parents at risk in future public health emergencies (e.g., disasters and disease outbreaks), comprehensive studies of risks occluding prior to such emergencies, are needed, spanning individual, relational, and contextual domains, relevant to both the parent and child(ren). Given there is likely a large set of potential predictors of risk, one methods to identifying the most salient risks for a given outcome is to utilise machine learning approaches. Machine learning methods can also be used to maximise prediction (as opposed to understanding causal explanations). Least Absolute Shrinkage and Selection Operator (LASSO) regression models (14) are one such approach which extend upon traditional regression models. Notably, such models (and their predictor coefficients) are not interpreted causally, but rather with the aim of identifying which parents are likely to be at risk in public health emergencies.

In this study we aimed to examine the extent to which pre-pandemic predictors (parent and child, individual, relational, contextual), that were prospectively measured when offspring were 1-year old (2012–2018), were associated with parent substance use (alcohol, tobacco, and illicit substances) during COVID-19 (mid-2020 and late-2021). We employed machine learning methods, specifically LASSO (Least Absolute Shrinkage and Selection Operator) penalised regression models, to identify the most predictive subset of indicators.

## 2 Methods

### 2.1 Participants

Participants were from the Australian Temperament Project (ATP), a 16-wave longitudinal study tracking the psychosocial development of young people from infancy to adulthood. In 1983, 67 Local Government Areas (LGAs) in the state of Victoria were randomly selected based on advice from the Australian Bureau of Statistics, to provide a representative sample of Victorian LGAs (15). Parents (Generation 1; G1) of every 4–8-month-old infant (Generation 2; G2) who visited one of the selected infant welfare centres between 22nd April and 6th May (1983) were invited to participate. Approximately 3,000 questionnaires were administered. The baseline sample consisted of 2,443 infants aged 4–8 months. Subsequently, families have participated via mail surveys approximately every 2 years until children were 19–20 years of age, and every 4 years thereafter (16).

The ATP Generation 3 (ATPG3) commenced in 2012, with recruitment of infant offspring (Generation 3; G3) of G2 participants and their partners. Identification of pregnancies occurred via participant contact every six months between 2012 and 2018 when participants were aged 29–35 years, representing the peak period of first births in Australia. Telephone or web-based surveys were conducted with parents at the third trimester of pregnancy, and at 8 weeks and 1-year postpartum, for which the 1-year postpartum data is used in the current study. Further information on sampling and procedures is available elsewhere (17) and a flowchart of the ATPG3 sample recruitment is presented in the supplementary material (17; Supplementary Figure S1). ATP Generation 3 Study protocols have been approved by the Royal Children's Hospital Human Research Ethics Committee (34185). Prior ATP waves were approved by the relevant committee at the time.

G2 participants in the ATPG3 study with one or more children were invited to complete a brief online survey about the impacts of the COVID-19 pandemic in: (1) 2020 between May and September, during the height of COVID-19 lockdowns (*n* = 515 parents; 60% female; 73% of the existing ATPG3 parent sample); and (2) 2021 between October and December (*n* = 488 parents; 59% female; 69% of existing ATPG3 parent sample). To assess bias due to attrition, ATPG3 participants were compared on characteristics collected at baseline (1983, at 4–8 months), including G2 sex, difficult temperament, and behaviour problems, as well as G1 education and country of birth. Compared to the full ATP sample, those who were screened for the ATPG3 study had marginally lower rates of G1s born overseas and G1s with less education. Those who participated in either of the COVID-19 surveys were representative of the ATPG3 sample. A total of 574 parents completed either COVID-19 assessment (59% female, 82% of the existing ATPG3 parent sample). To be included in the current sample, participants needed to have further provided data at the 1-year postpartum survey. The resulting sample included 560 G2 parents (59% female).

### 2.2 Measures

#### 2.2.1 Substance use during the COVID-19 pandemic

Frequency of alcohol (item: “Alcohol”), tobacco (item: “Cigarettes or other tobacco?”), cannabis [item: “Cannabis (e.g., marijuana joint, blunt, pipe, bong)”], and other illicit substances (item: “Other drugs including opiates, heroin, or narcotics cocaine, crack, amphetamine, methamphetamine, hallucinogens, or ecstasy”) in the past two weeks were assessed at both COVID-19 waves. Response options were: 1 = “Not at all”, 2 = “1 or 2 days a week”, 3 = “3 or 4 days a week”, 4 = “Almost every day”. Responses to cannabis and other illicit substances were combined to indicate any illicit use. Binary variables were derived to indicate potentially harmful levels of use. For alcohol, derived binary variables indicated drinking around half the week or more (i.e., reporting at least “3 or 4 days a week”). For tobacco and illicit substance use derived binary variables indicated any use (i.e., reporting at least “1 or 2 days a week”). Given the relatively low prevalence of substance use (particularly tobacco and illicit substance use) and consistent pattern at each COVID-19 timepoint [alcohol: 32% (t1), 29% (t2); tobacco: 7% (t3), 8% (t2); illicit substance use: 3% (t1), 2% (t2)], variables were combined across COVID-19 timepoints.

#### 2.2.2 Pre-COVID-19 postpartum (1-year) predictors

A summary of all pre-COVID-19 postpartum (1-year) predictor variables is presented in Table 1 (see Supplementary Table S1 for further details). Measures included: G3 individual factors (i.e., sex, birth weight, gestational age, number of siblings, health, behaviour problems and competencies, temperament) and relational factors (i.e., hours in care with family members and non-family members); G2 individual factors (i.e., sex, mental health, substance use), relational factors (i.e., parenting style, social support, parent-infant bonding), and contextual factors (i.e., stressful life events, marital status, employment status, education level, financial situation). G2 participant responses were used for all measures, however, for measures related to the G3 and those reflecting the G2 context, partner responses supplemented missing participant responses.

**Table 1:** Descriptive statistics for postpartum (1-year) predictor variables.

Measures	*n*	%	M	SD	95% CI	*n*
Infant (G3) characteristics						
Individual						
Female	295	53%			(49, 57)	560
Birth weight			3.4	0.5	(3.4, 3.5)	549
Gestational age			39.1	1.7	(39.0, 39.2)	552
Number of siblings			1.0	0.9	(0.9, 1.1)	560
Health			3.7	0.6	(3.6, 3.7)	552
Behaviour problems			7.3	4.3	(6.9, 7.7)	550
Behaviour competencies			15.8	2.8	(15.5, 16.0)	550
Approach			13.7	4.6	(13.3, 14.1)	549
Cooperation			18.7	4.3	(18.4, 19.1)	549
Persistence			13.1	3.5	(12.8, 13.4)	550
Rhythmicity			9.4	2.9	(9.2, 9.6)	551
Distractibility			16.6	2.6	(16.4, 16.8)	549
Reactivity			29.3	4.5	(28.9, 29.7)	550
Relational						
Family care hours			4.6	10.3	(3.7, 5.5)	552
Non-family care hours			4.8	9.8	(4.0, 5.6)	552
Parent (G2) characteristics						
Individual						
Female	330	59%			(55, 63)	560
Depression			1.2	1.9	(1.0, 1.4)	490
Anxiety			0.7	1.5	(0.6, 0.9)	490
Stress			3.6	3.1	(3.3, 3.8)	490
Alcohol use (≥3–4 days per week)	98	20%			(17, 24)	489
Tobacco use (≥1–2 day per week)	61	13%			(10, 16)	488
Illicit use (≥1–2 day per week)	24	5%			(3, 7)	489
Relational						
Parenting self-efficacy			3.2	0.8	(3.1, 3.3)	489
Parental warmth			4.4	0.5	(4.3, 4.4)	490
Parental hostility			1.8	0.5	(1.8, 1.9)	490
Parental anxiety			3.0	0.6	(3.0, 3.1)	490
Social support			4.4	0.5	(4.4, 4.4)	366
Maternal-infant bonding†			80.9	6.4	(80.2, 81.6)	329
Paternal-infant bonding†			76.2	7.6	(75.0, 77.4)	157
Contextual						
Stressful life events			0.4	0.7	(0.3, 0.4)	491
Married/de facto	469	86%			(83, 89)	543
Paid employment	447	80%			(76, 83)	560
Education			5.5	1.9	(5.3, 5.6)	478
Financial stress			0.9	0.9	(0.8, 1.0)	549

^†^Scored separately for mothers and fathers.

### 2.3 Statistical analysis

All analyses were conducted in Stata 17 (18). Missing data within the analysis sample ranged from 0% to 35%. Multiple imputation was used to handle missing data. Twenty complete datasets were imputed, based on a multivariate normal model (19). Binary variables were imputed as continuous variables and then back transformed with adaptive rounding following imputation (20). This approach to imputation has been shown to perform similarly to imputation models using chained equations (19). Estimates were obtained by pooling results across the 20 imputed datasets using Rubin's rules (21).

First, descriptive statistics (mean/SD, *n*/%) were provided for all variables included in the current study using the non-imputed data. Next, predictors [k = number of predictors] were identified using Least Absolute Shrinkage and Selection Operator (LASSO) penalised logistic regression models, for which each outcome was regressed onto the 33 predictors, in multivariable models. In comparison to traditional regression methods, the LASSO models shrink the size of coefficients by applying a penalty factor and, in turn, retain the most important predictor variables (i.e., coefficients greater than zero) (14). Ten-fold cross-validation was used to select the penalty strength which minimises the cross-validation function (i.e., to identify the best fitting model). Predictive performance was assessed using the area under the Receiver Operating Characteristic (ROC) Curve (AUC; ≥0.7 is acceptable, ≥0.8 is excellent, and ≥0.9 is outstanding) (22). Given that the LASSO model has not been embedded within Stata's imputation commands, models were run on each imputed dataset and then coefficients were averaged if a predictor was retained in at least 80% of imputed datasets (with AUC values averaged across all datasets). Traditional logistic regressions models were used to estimate the association between each pre-COVID-19 postpartum predictor and substance use during the COVID-19 pandemic (adjusting for the time since the 1-year survey). Of note, while confidence intervals are estimated from traditional regression models, no confidence intervals are estimated as part of the LASSO model – these models aim to maximise prediction, not quantify the uncertainty of individual parameter estimates. All models included adjustment for time since the 1-year survey and the start of the COVID-19 surveys (12 May 2020) to account for variation in the timing between assessments. All continuous variables were z-scored prior to analysis.

## 3 Results

### 3.1 Descriptive information

During the COVID-19 pandemic, 39% (95% CI = 35, 43) of parents reported using alcohol at least 3–4 days per week [79% (95% CI = 76, 83) used at least 1 day per week], 12% (95% CI = 9, 15) reported using any tobacco, and 6% (95% CI = 4, 8) reported use any illicit substance use. Patterns of use were similar or somewhat higher in males [alcohol: 46% (95% CI = 39, 52); tobacco: 14% (95% CI = 10, 19); illicit substance use: 7% (95% CI = 4, 11)] relative to females [alcohol: 34% (95% CI = 29, 39); tobacco: 10% (95% CI = 7, 14); illicit substance use: 6% (95% CI = 4, 9)] participants. A descriptive summary of all postpartum predictor variables is presented in Supplementary Table S1.

### 3.2 Postpartum predictors of substance use during the COVID-19 pandemic

Associations between pre-pandemic, postpartum predictors and substance use during the COVID-19 pandemic are presented in Table 2 for LASSO and traditional logistic regression models.

**Table 2:** Penalised and traditional logistic regression models examining relationships between COVID-19 substance use and pre-COVID-19 postpartum (1-year) predictors (*N* = 560).

Measures	Alcohol use			Tobacco use			Illicit use		
	OR_L_	OR	95% CI	OR_L_	OR	95% CI	OR_L_	OR	95% CI
Infant (G3) characteristics									
Individual									
Female	–	0.73	(0.52, 1.03)	–	1.05	(0.62, 1.78)	–	1.31	(0.64, 2.65)
Birth weight	–	0.99	(0.83, 1.18)	–	0.70	(0.54, 0.91)	–	0.85	(0.61, 1.20)
Gestational age	–	1.06	(0.90, 1.27)	–	0.83	(0.65, 1.07)	–	1.20	(0.83, 1.75)
Number of siblings	–	0.83	(0.70, 0.99)	–	0.98	(0.75, 1.28)	–	0.97	(0.69, 1.38)
Health	–	0.99	(0.84, 1.18)	–	0.97	(0.74, 1.26)	–	1.15	(0.78, 1.69)
Behaviour problems	–	1.09	(0.92, 1.30)	–	1.14	(0.88, 1.48)	–	1.20	(0.86, 1.67)
Behaviour competencies	–	0.89	(0.75, 1.06)	1.73	1.49	(1.11, 1.98)	–	1.28	(0.89, 1.85)
Approach	–	0.91	(0.76, 1.08)	–	0.78	(0.59, 1.03)	–	1.06	(0.75, 1.50)
Cooperation	–	1.14	(0.96, 1.36)	–	1.00	(0.77, 1.31)	–	0.95	(0.67, 1.35)
Persistence	–	1.06	(0.90, 1.26)	–	0.87	(0.66, 1.14)	–	0.84	(0.58, 1.20)
Rhythmicity	–	0.96	(0.81, 1.14)	–	1.05	(0.81, 1.36)	–	1.10	(0.78, 1.54)
Distractibility	–	0.96	(0.80, 1.14)	0.87	0.78	(0.60, 1.02)	–	0.92	(0.65, 1.31)
Reactivity	–	0.96	(0.81, 1.14)	1.29	1.21	(0.92, 1.58)	–	1.07	(0.76, 1.51)
Relational									
Family care hours	–	0.89	(0.74, 1.08)	0.85	0.72	(0.51, 1.03)	–	0.78	(0.49, 1.25)
Non-family care hours	–	1.02	(0.86, 1.21)	0.88	0.93	(0.70, 1.24)	–	1.08	(0.78, 1.49)
Parent (G2) characteristics									
Individual									
Female	–	0.61	(0.43, 0.86)	–	0.60	(0.35, 1.02)	–	0.83	(0.41, 1.67)
Depression	–	0.93	(0.77, 1.13)	–	1.03	(0.79, 1.33)	–	1.22	(0.91, 1.65)
Anxiety	–	1.07	(0.88, 1.30)	–	0.93	(0.70, 1.24)	–	1.00	(0.70, 1.45)
Stress	–	1.13	(0.94, 1.35)	0.81	0.75	(0.55, 1.03)	–	1.01	(0.69, 1.46)
Alcohol use (≥3/4days/week)	2.03	7.97	(4.77, 13.32)	–	1.52	(0.76, 3.06)	–	1.93	(0.86, 4.31)
Tobacco use (any)	–	1.29	(0.76, 2.19)	4.21	69.16	(29.61, 161.55)	1.44	8.22	(3.63, 18.60)
Illicit use (any)	–	2.41	(1.05, 5.51)	–	5.79	(2.15, 15.59)	1.60	18.97	(7.41, 48.57)
Relational									
Parenting self-efficacy	–	1.05	(0.87, 1.27)	–	0.97	(0.71, 1.31)	–	1.17	(0.79, 1.73)
Parental warmth	–	0.88	(0.73, 1.05)	–	1.19	(0.87, 1.62)	–	0.77	(0.55, 1.09)
Parental hostility	–	1.15	(0.96, 1.38)	–	0.93	(0.70, 1.25)	–	1.19	(0.83, 1.72)
Parental anxiety	–	1.07	(0.89, 1.29)	–	1.01	(0.75, 1.35)	–	0.87	(0.59, 1.28)
Social support	–	0.97	(0.77, 1.24)	–	1.06	(0.74, 1.52)	–	1.04	(0.67, 1.61)
Parent-infant bonding	0.92	0.77	(0.64, 0.93)	–	1.23	(0.88, 1.71)	–	0.83	(0.58, 1.20)
Contextual									
Stressful life events	–	0.85	(0.71, 1.03)	–	1.04	(0.79, 1.39)	–	1.18	(0.85, 1.63)
Married/de facto	–	0.76	(0.46, 1.25)	0.61	0.29	(0.15, 0.54)	–	0.44	(0.19, 0.98)
Paid employment	–	1.20	(0.78, 1.85)	0.86	0.48	(0.27, 0.85)	–	0.64	(0.29, 1.40)
Education	–	1.05	(0.88, 1.27)	0.78	0.56	(0.42, 0.75)	–	0.92	(0.63, 1.36)
Financial stress	–	1.07	(0.90, 1.27)	–	1.36	(1.06, 1.73)	–	1.02	(0.73, 1.43)

OR_L_, odds ratio from LASSO model; traditional regression coefficients at *p* < 0.05 bolded.

For alcohol use during the pandemic, LASSO models (AUC=0.72) identified two predictors (k = 2): prior alcohol use (OR = 2.03) and parent-infant bonding (OR = 0.92). These associations were supported by evidence from traditional regression models: prior alcohol use (OR = 7.97, 95% CI = 4.77, 13.32) and parent-infant bonding (OR = 0.77, 95% CI = 0.64, 0.93).

For tobacco use during the pandemic, LASSO models (AUC = 0.96) identified ten (k = 10) predictors including G3 behaviour competencies (OR = 1.73), distractibility (OR = 0.87), reactivity (OR = 1.29), and hours at in family (OR = 0.85) and non-family care (OR = 0.88), and G2 stress (OR = 0.81), tobacco use (OR = 4.21), marital status (OR = 0.61), employment (OR = 0.86), and education (OR = 0.78). Five of these predictors were also evidenced in traditional regression models: behaviour competencies (OR = 1.49, 95% CI = 1.11, 1.98), tobacco use (OR = 69.16, 95% CI = 29.61, 161.55), marital status (OR = 0.29, 95% CI = 0.15, 0.54), paid employment (OR = 0.48, 95% CI = 0.27, 0.85), and education level (OR = 0.56, 95% CI = 0.42, 0.75).

For illicit substance use during the pandemic, LASSO models (AUC=0.78) identified two predictors (k = 2): postpartum tobacco use (OR = 1.44) and illicit substance use (OR = 1.60). These were also identified in traditional regression models: tobacco use (OR = 8.22, 95% CI = 3.63, 18.60) and illicit substance use (OR = 18.97, 95% CI = 7.41, 48.57).

## 4 Discussion

Here we draw on prospective data to examine pre-pandemic predictors of parent substance use during the COVID-19 pandemic in Australia, which experienced some of the longest periods of public health restrictions in the world throughout 2020 and 2021. Most prominently, pre-pandemic substance use was identified as the strongest predictor for all substance types during COVID-19 (alcohol, tobacco and illicit substance use); a finding which is consistent with prior literature that has identified strong continuities in substance use behaviour into parenthood (23).

Further, pre-pandemic parent-infant bonding and tobacco use were identified in LASSO models as the only other predictors of parental alcohol and illicit drug use, respectively, during the pandemic. The association with parent-infant bonding is consistent with previously observed patterns of poorer quality parent-infant bonding among parents who report substance use (24). One explanation is that infants of parents with stronger bonds have better behavioural and developmental outcomes (25), which is linked to reduced parenting stress (26) and lower likelihood of parental alcohol consumption. Further to this, we note that the association between pre-pandemic tobacco use and COVID-19 illicit drug use is in line with the high prevalence of tobacco co-use identified among people using illicit substances (27).

A more complex predictive model emerged for tobacco use during the pandemic, with family factors spanning both child (individual and relational) and parent (individual and contextual) characteristics emerging as important predictors of tobacco use. Associations were consistent across several contextual factors (e.g., marital status, employment, and education level). Current findings support previous work identifying socioeconomic disadvantage as a key predictor of tobacco use (28) and reflect patterns of risk identified in parents assessed during the pandemic (12). While population-wide strategies such as media campaigns, tax increases, and smoke-free legislation have been effective in reducing smoking across socioeconomic groups (29), current findings suggest that parents impacted by greater disadvantage would benefit from more targeted support around smoking cessation (e.g., social media targeting based on geographic location).

Further, a range of family characteristics, spanning child and parent factors, were identified in risk profiles for tobacco use during the pandemic. Need a summary sentence here. Interestingly, while the direction of the reactivity finding supports prior risk associations between parental smoking and negative child outcomes (30), several associations were in unexpected directions (e.g., a risk association for behaviour competencies and a protective association for parent stress). Given the non-causal modelling approach in the current study (i.e., predictive LASSO models) further research is required to clarify these relationships. The current findings also suggest that the identification of predictors of tobacco use behaviours requires a multifaceted approach.

### 4.1 Implications

The strong prediction of pandemic substance use by pre-pandemic patterns of substance use highlights the need for treatment services and early intervention to prevent use from becoming entrenched. The COVID-19 pandemic has profoundly changed the way in which people access healthcare. In Australia, despite high rates of substance use (31), rates of treatment uptake remain low and delays in treatment-seeking are substantial (32, 33). Telehealth provides a promising strategy to increase earlier engagement with alcohol and substance use treatment, by providing a format of treatment delivery that offers a sense of privacy and anonymity, which can help to overcome treatment barriers related to fear of shame and stigma, as well as structural barriers such as constraints related to travel time and need for childcare. Such models of treatment may be filling a gap in service provision for women who often perform significant childcare duties and who may be less able to access traditional in-person services due to childcare responsibilities (34, 35). Online treatment/app interventions could be another option.

For parents of young families, as well as those planning families, routine prenatal visits provide an opportunity for alcohol and drug screening, provision of information on substance use risks and supports, and where suitable, referral and intervention, which may in tuen increase engagement with alcohol and other drug support services (as well as reduce risk of harm to infants, e.g., FASD, family violence) (36). However, it also important to recognise that, for many people, prenatal substance use behaviours have a history of continuity from adolescence and into the young adult period, well before conception (23). This suggests that universal monitoring and intervention efforts, extending earlier in the life course, are likely to be beneficial in disrupting future patterns of continuity into the parenting period.

### 4.2 Limitations

In mature cohort studies there are important sources of selection, measurement, and confounding bias that require consideration. Although multiple imputation was used to minimize missing data bias, it is likely that there was some selective attrition of the most vulnerable individuals, such as those with high levels of substance use. Further, parents who had a child prior to the recruitment period (parents aged 29–35 years) are excluded, unless they also went on to have another child in this period. Additionally, as all measures are self-reported, there is a risk of shared method variance and social desirability bias. To mitigate these issues, one option could be to incorporate data from additional sources, such as family members or peers, or employ approaches like the Randomized Response Technique (37) into future research methods. Further, substance use outcomes were defined based only on frequency, and therefore do not capture aspects of substance use related to quantity or disorder, which may have differential predictive profiles. The strength of associations may also be impacted by the variation in time (i.e., less than one year up to nine years) between completing the 1-year postpartum survey and the COVID-19 survey, although accounted for analytically. Further, the relatively small sample size may have meant that the study was underpowered to detect weaker associations of interest (that might be expected across long periods of time), as indicated by the imprecise confidence intervals. As such, the pattern of results are likely to be conservative in nature. Finally, while the current aims were not causal in nature, future work could examine causal pathways to determine appropriate intervention opportunities.

## 5 Conclusions

In our study, the most important predictor of parental substance use during the COVID-19 pandemic was prior substance use, pointing to the importance of enriching support to those with histories of substance use. Given the strong continuity in substance use behaviours, public health policy should consider improved access to substance use support and treatment services, particularly in times of public health emergencies. However, to comprehensively prepare for future public health emergencies, continued efforts are needed to identify and protect against broader social inequalities and vulnerabilities. This was evident in the more complex predictive models that emerged for parental tobacco use. Together, findings suggest that beyond patterns of substance use continuity, approaches to targeting interventions, particularly for tobacco use, likely requires efforts which consider a range of individual, relational and contextual factors.

## Data Availability

The datasets presented in this article are not readily available because Ethics approvals for this study do not permit these potentially re-identifiable participant data to be made publicly available. Enquires about collaboration are possible through our institutional data access protocol: https://lifecourse.melbournechildrens.com/data-access/. The current institutional body responsible for ethical approval is The Royal Children's Hospital Human Research Ethics Committee. Requests to access the datasets should be directed to Craig Olsson, craig.olsson@deakin.edu.au.
