# Supplementary material for: Pre-pandemic predictors of parental substance use during COVID-19
Source: Front Child Adolesc Psychiatry. 2025 Oct 7;4:1587146. doi: 10.3389/frcha.2025.1587146 (PMC12537729; doi:10.3389/frcha.2025.1587146)
Supplement: Supplementary file 1 [file Datasheet1.docx]

| 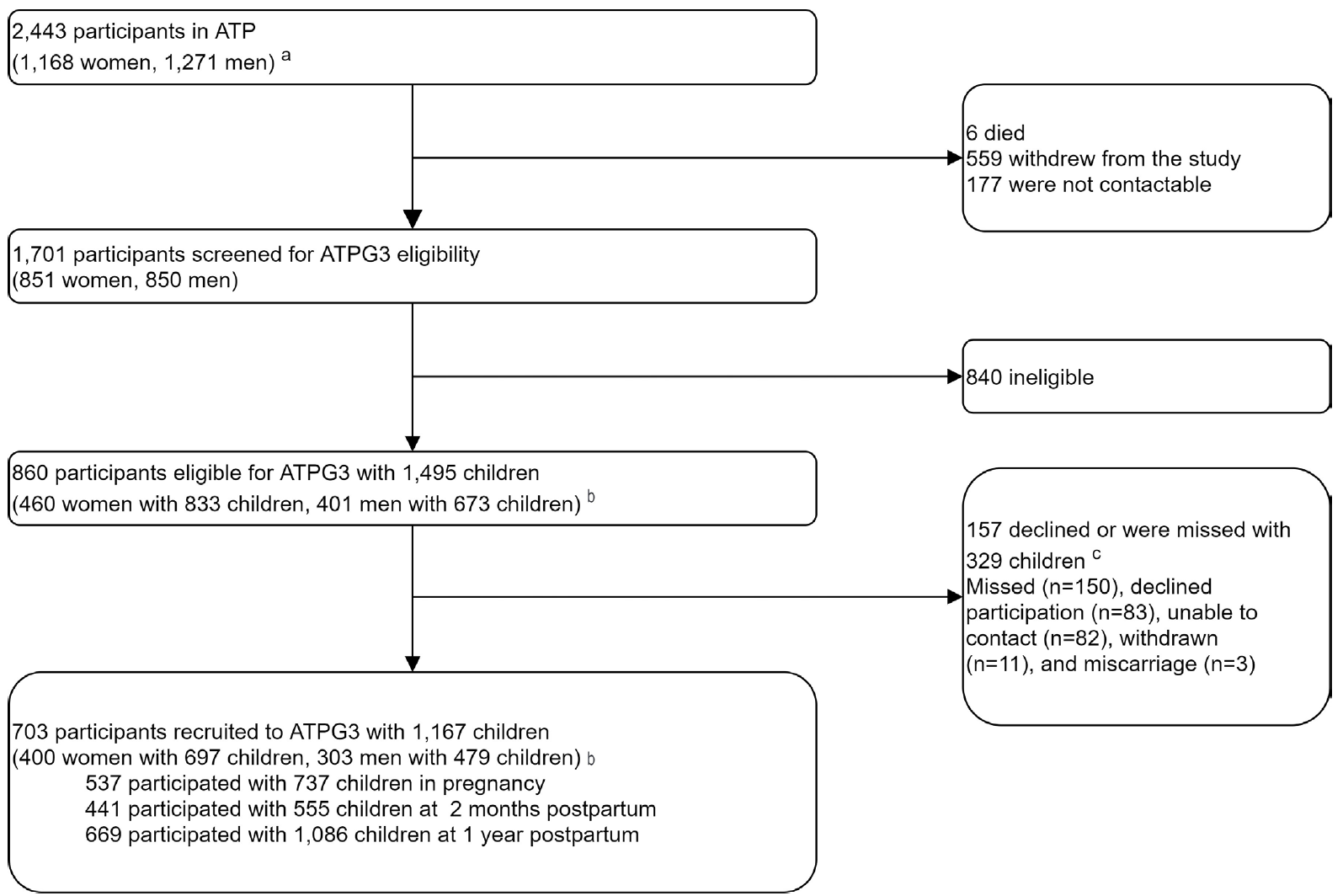 |
| --- |
| Figure S1. Reproduced from Olsson et al. (2022). Licensed under CC BY-NC.  Flowchart of The Australian Temperament Generation 3 Study (ATPG3) participants from recruitment to 1 year postpartum data collection. Note: study entry could occur during any perinatal wave. ATPG3 eligibility defined as all children younger than 18 months identified during screening (December 2011 to September 2018); until mid-2013 recruitment included children up to 3 years of age. ^a^Four ATP participants missing sex data. ^b^The number of children by women and men does not sum to total number of children because,9 of 1167 children recruited came from five ATP couples. ^c^One child participated in ATPG3 with the other ATP parent. |

| Table S1. Measurement and descriptive summary of pre-COVID-19 postpartum (1-year) predictors | | | | | | |
| --- | --- | --- | --- | --- | --- | --- |
|  | Description | Scoring | n | % | M | SD |
| Infant (G3) characteristics |  |  |  |  |  |  |
| Individual |  |  |  |  |  |  |
| Sex (female) | Child sex | 0=Male, 1=Female | 295 | 53% |  |  |
| Birth weight | Birth weight | Kilograms |  |  | 3.4 | 0.5 |
| Gestational age | Gestation age | Weeks |  |  | 39.1 | 1.7 |
| Number of siblings | Number of siblings | Count |  |  | 1.0 | 0.9 |
| Health | Compared to other children his/her age, would you say (names) health is | 0=Very poor to 4=Excellent |  |  | 3.7 | 0.6 |
| Behaviour |  |  |  |  |  |  |
| Behaviour problems | Behavioural problems and competencies; BITSEA (Briggs-Gowan et al. 2004) | 0=Not true/rarely to 2=Very true/often |  |  | 7.3 | 4.3 |
| Behaviour competencies |  |  |  |  | 15.8 | 2.8 |
| Temperament |  |  |  |  |  |  |
| Approach | Approach, cooperation, persistence, rhythmicity, distractibility, reactivity; STST (Sewell et al. 1988) | 1=Almost never to 6=Almost always |  |  | 13.7 | 4.6 |
| Cooperation |  |  |  |  | 18.7 | 4.3 |
| Persistence |  |  |  |  | 13.1 | 3.5 |
| Rhythmicity |  |  |  |  | 9.4 | 2.9 |
| Distractibility |  |  |  |  | 16.6 | 2.6 |
| Reactivity |  |  |  |  | 29.3 | 4.5 |
| Relational |  |  |  |  |  |  |
| Hours in care |  |  |  |  |  |  |
| Family care hours | Hours in care with other family members and non-family members | Hours |  |  | 4.6 | 10.3 |
| Non-family care hours |  |  |  |  | 4.8 | 9.8 |
| Parent (G2) characteristics |  |  |  |  |  |  |
| Individual |  |  |  |  |  |  |
| Sex (female) | Parent sex | 0=Male, 1=Female | 330 | 59% |  |  |
| Mental health |  |  |  |  |  |  |
| Depression | Depression, anxiety, stress; DASS-21 (Lovibond and Lovibond 1995) | 0=Not at all to 3=Very much, or most of the time |  |  | 1.2 | 1.9 |
| Anxiety |  |  |  |  | 0.7 | 1.5 |
| Stress |  |  |  |  | 3.6 | 3.1 |
| Substance use |  |  |  |  |  |  |
| Alcohol use (≥3-4 days per week) | Past month alcohol, tobacco, and illicit substance use | 0=<3-4 days/week, 1=≥3-4 days/week; | 98 | 20% |  |  |
| Tobacco use (≥1-2 day per week) |  | 0=no use, 1=any use. | 61 | 13% |  |  |
| Illicit use (≥1-2 day per week) |  | 0=no use, 1=any use. | 24 | 5% |  |  |
| Relational |  |  |  |  |  |  |
| Parenting self-efficacy | Overall as a parent, do you feel that you are | 0=Not very good at being a parent to 4=A very good parent |  |  | 3.2 | 0.8 |
| Parenting style |  |  |  |  |  |  |
| Parental warmth | Warmth, hostility, and anxiety; From LSAC (Zubrick, Lucas and Westrupp 2014) | 1=Never/almost never to 5=Always/almost always |  |  | 4.4 | 0.5 |
| Parental hostility |  |  |  |  | 1.8 | 0.5 |
| Parental anxiety |  |  |  |  | 3.0 | 0.6 |
| Social support | Social support; MSSS (Webster et al. 2000) | 1=Never to 5=Always |  |  | 4.4 | 0.5 |
| Parent-infant bonding |  |  |  |  |  |  |
| Maternal-infant bonding | Overall bonding; MPAS/PPAS (Condon and Corkindale 1998, Condon, Corkindale and Boyce 2008) | Varied Likert responses, higher scores=higher bonding |  |  | 80.9 | 6.4 |
| Paternal-infant bonding |  |  |  |  | 76.2 | 7.6 |
| Contextual |  |  |  |  |  |  |
| Stressful life events | Number of bad life events | Count |  |  | 0.4 | 0.7 |
| Marital status (married/de-facto) | Current marital status | 0=Not currently married/de facto, 1=Currently married/de facto | 469 | 86% |  |  |
| Employment status (in paid) | Employment status | 0=Not in paid employment, 1=In paid employment | 447 | 80% |  |  |
| Education level | Highest level of education | 0=Year 8/9/10; or equivalent to 8= Postgrad degree |  |  | 5.5 | 1.9 |
| Financial situation | Current financial situation | 0=Living comfortably to 4=Finding it very difficult |  |  | 0.9 | 0.9 |
| Note: BITSEA=Brief Infant-Toddler Social and Emotional Assessment; STST=Short Temperament Scale for Toddlers; DASS=Depression Anxiety Stress Scale: Short Form; LSAC=the Longitudinal Study of Australian Children; MSSS=Maternity Social Support Scale; MPAS=Maternal Postpartum Attachment Scale; PPAS=Paternal Postpartum Attachment Scale | | | | | | |

Briggs-Gowan, Margaret J., Carter, Alice S., Irwin, Julia R., Wachtel, Karen, and Cicchetti, Domenic v., ‘The Brief Infant-Toddler Social and Emotional Assessment: Screening for Social-Emotional Problems and Delays in Competence’, *Journal of Pediatric Psychology*, 29/2 (2004), 143–55

Condon, John T., and Corkindale, Carolyn J., ‘The Assessment of Parent-to-Infant Attachment: Development of a Self-Report Questionnaire Instrument’, *Journal of Reproductive and Infant Psychology*, 16/1 (1998), 57–76

Condon, John T., Corkindale, Carolyn Jennifer, and Boyce, Phillip, ‘Assessment of Postnatal Paternal-Infant Attachment: Development of a Questionnaire Instrument’, *Journal of Reproductive and Infant Psychology*, 26/3 (2008), 195–210

Lovibond, S. H., and Lovibond, P. F., *Manual for the Depression Anxiety Stress Scales*, *Psychology Foundation of Australia*, 1995

Sewell, J., Oberklaid, F., Prior, M., Sanson, A., and Kyrios, M., ‘Temperament in Australian Toddlers’, *Journal of Paediatrics and Child Health*, 24/6 (1988), 343–45 <https://onlinelibrary.wiley.com/doi/10.1111/j.1440-1754.1988.tb01385.x>

Webster, Joan, Linnane, John WJ, Dibley, Linda M, Hinson, Janis K, Starrenburg, Suzanne E, and Roberts, Janice A, *Measuring Social Support in Pregnancy: Can It Be Simple and Meaningful? Postnatal Depression and 300 without Risk Factors)*, *BIRTH*, 2000, xxvii

Zubrick, Stephen R, Lucas, Nina, and Westrupp, Elizabeth, ‘Parenting Measures in the Longitudinal Study of Australian Children: Construct Validity and Measurement Quality, Waves 1 to 4’, 2014 <https://www.researchgate.net/publication/264533994>
